# Stationary Fatigue Resistance of Various Rotary Instruments with Different Alloys after Preparing Three Root Canals

**DOI:** 10.14744/eej.2021.93685

**Published:** 2021-11-15

**Authors:** Afzal ALI, Nihat Umut GÖZEN, Abdul Kerim KUL, Naveen CHHABRA

**Affiliations:** 1. Department of Conservative Dentistry and Endodontics, Pacific Dental College and Hospital, Rajasthan, India; 2. Department of Endodontics, Faculty of Dentistry, Istanbul Health Sciences University, Istanbul, Turkey; 3. Department of Conservative Dentistry and Endodontics, Faculty of Dental Sciences, Narsinhbhai Patel Dental College and Hospital, Sankalachand Patel University, Visnagar, Gujarat, India

**Keywords:** Nickel-Titanium (NiTi), root canal therapy, stationary fatigue, trunatomy

## Abstract

**Objective:**

This study aimed to evaluate the stationary fatigue resistance of three endodontic instrument systems after preparing three root canals with different curvatures and comparing them with unused instruments.

**Methods:**

A total of 60 endodontic files from 3 instrument systems, TruNatomy (TRN), HyFlex CM (HFC) and Revo-S (RS), were selected for this study. These systems were divided into 2 groups: Group I (Used files) and Group II (Unused files). Each group was further divided into 3 subgroups, each containing 10 TRN (#26/v.04), 10 HFC (#25.04) and 10 RS (#25/.04) rotary files. The group I instruments were used for shaping the root canals of extracted third molars, while the group II instruments were not used for canal shaping procedures. Both Group I and Group II instruments were subjected to stationary cyclic fatigue testing at simulated body temperature (37±0.5°C) using a stainless-steel block with an artificial canal. The number of cycles to failure (NCF), the length of fractured instruments and the preparation time were recorded. The statistical analysis was performed using two-way ANOVA and Tukey post-hoc tests with a 95% confidence interval (P=0.05).

**Results:**

NCF was signiﬁcantly affected by the instrument type and whether the instrument was unused or used (P<0.001). The Group II instruments were more resistant to stationary cyclic fatigue than Group I (P<0.05). HFC instruments were most resistant to stationary fatigue among all tested conditions, followed by TRN and RS instruments. Canal preparation with TRN was significantly faster than with HFC and RS. During preparation, no file was fractured. A statistically significant difference (P<0.05) was observed in the mean length of the fractured instruments among used instrument groups.

**Conclusion:**

The stationary cyclic fatigue resistance of HFC instruments were significantly greater than that of the TRN and RS instruments (P<0.05). TRN was faster in shaping the root canals than other instruments tested in the study.

## Introduction

One of the potential problems during the root canal treatment is the fracture of nickel-titanium (NiTi) instruments. The main mechanisms of such fracture are torsional stress and cyclic fatigue failure (1). Torsional failure occurs when the tip of the rotary instrument is bound within the root canal, and the shank continues to rotate, with the tip separating from the file when the torque exceeds the instrument’s elastic limit. Cyclic fatigue occurs due to repeated cycles of tension and compression, leading to fracture of the instruments. Cyclic fatigue testing can be categorised as dynamic or static. In dynamic cyclic fatigue testing, the instrument moves back and forth into the simulated root canal with a more uniform distribution of the stresses along the instrument shaft. In contrast, no such movement occurs in static cyclic fatigue testing, and hence stresses are concentrated in a single area (2).

**HIGHLIGHTS**

• The unused instruments (Group II) showed higher NCF as compared to the used ones (Group I) (P<0.05).
• The HFC instruments showed better resistance to fracture than TRN and Revo-S in-struments.
• All the instruments were safe while preparing the root canals during the ex-vivo usage.
• Within test conditions, the TRN instruments shaped the canals in a faster way.
• It should be kept in mind that the file can be broken faster when treating multiple teeth.

Stationary fatigue failure may occur at the highest degree of root canal curvature (3-5). To increase the fracture resistance of the NiTi instruments, the manufacturers have altered the instrument’s properties by surface or thermal treatment, cross-section design, and altering the alloy ratios (6).

HyFlex CM (HFC) (Coltène-Whaledent, Altstätten, Switzerland) is designed using controlled memory alloy. It is produced by a thermal treatment that includes both heating and cooling processes. HFC instruments have 2 different cross-sections: 6% tapered instruments with a triangular cross-section and 4% tapered instruments with a quadrangular cross-section. HFC has been reported to have increased flexibility with 300% higher cyclic fatigue resistance than the original alloy. As a result, the instrument offers improved canal shaping and better maintenance of the original root canal curvature (7, 8). Topçuoğlu et al. reported higher cyclic fatigue resistance with HFC as compared to OneShape, ProTaper Universal and ProTaperNext instruments in the apical curvature of an artificial canal (9).

Revo-S (RS) (Micro Mega Besancon, France), manufactured using conventional NiTi alloy and has a variable cross-section and three cutting edges in all parts of the file. The asymmetrical section initiates a snake-like movement of the file inside the root canal. It consists of a set of 6 files. Unlike other RS instruments, Revo-SC2 (#25/.04) instrument design is not off-centred (10). Studies reported lower NCF values with RS instruments as compared to ProTaper Next X2, OneShape and HFC (11).

Recently, TruNatomy (TRN) (Dentsply Sirona, Ballaigues, Switzerland) heat-treated NiTi instruments have been developed. It consists of 5 specific instruments. It has an off-centred parallelogram cross-section, which provides space for additional debris removal. While conventional NiTi instruments have been manufactured using 1.2 mm NiTi wire, TRN has been manufactured using a NiTi wire size of 0.8 mm. Hence the maximum flute diameter is 0.8 mm. The manufacturer claims to preserve tooth structure and canal geometry, especially in the severely curved canal, because of its regressive slim taper, less shape memory, and special heat treatment of the NiTi alloy. The regressive taper design provides a reducing taper from the tip to the shaft, thereby improving the instrument’s flexibility (7, 12-14).

Repetitive use of endodontic instruments often results in microcrack formation, resulting in instrument failure (6). TRN instruments are intended for single use. The manufacturer claims that the mechanical characteristics of TRN allow the preparation of at least four root canals. It is highly possible that, after preparing certain root canals, the instrument may undergo fatigue and possibly get separated during the preparation of the additional root canals.

To our best knowledge, fewer limited researches are available comparing the stationary fatigue resistance of TRN with other currently available endodontic instruments. Thus, the present study was aimed to compare the stationary fatigue resistance of TRN and the existing heat-treated NiTi instruments (HFC, RS) of similar dimensions after their ex-vivo usage at simulated body temperature. The null hypothesis for the present study stated that there would be no significant differences among the groups in terms of stationary fatigue and preparation time.

## Materials and Methods

Prior institutional Ethical Committee (PDCH/20/EC-228) approval was obtained for this *in-vitro* study.

### a) Specimen population:

Only extracted third molars were included in the present study due to the frequent occurrence of variable degrees of canal curvatures and easy availability owing to their impacted nature. Teeth with calcified root canals, internal/external root resorption, tooth fractures, any foreign material in the canals (gutta-percha, fractured instruments etc.) and canals having an initial apical diameter greater than #10 K-file were excluded from the study.

### b) Instrument’s selection and groups:

Group I (Used rotary instruments) (n=30) – Group I was further subdivided into Ia:TRN (n=10), Ib:HFC (n=10) and Ic:RS (n=10). These rotary instruments were used for shaping the root canals of extracted third molars.

Group II (Unused rotary instruments) (n=30) - Group II was further subdivided into IIa: TRN (n=10), IIb: HFC (n=10) and IIc: RS (n=10) and were directly used for the cyclic fatigue testing.

### c) Experimental procedure (*Ex-vivo* usage):

**Figure 1.**
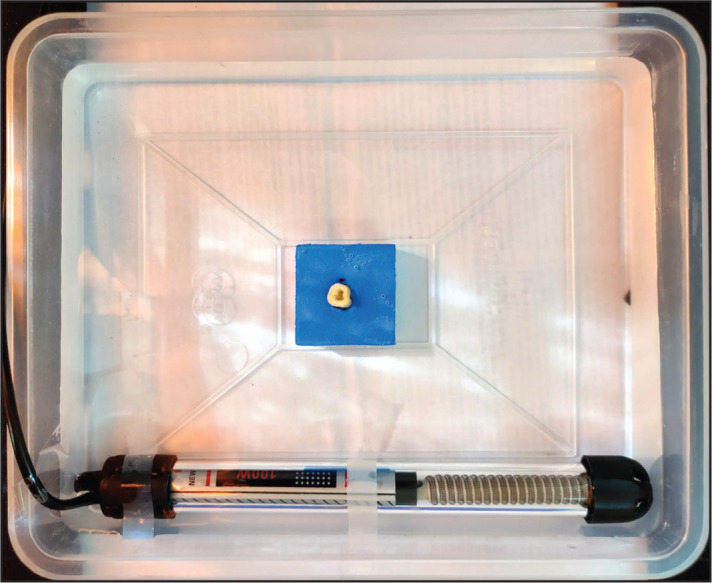
Shows the preparation of root canal at simulated body temperature

The teeth were decoronated to obtain a standardised working length of 18±0.5 mm, and access was established to the root canals. Size #10K-file (Dentsply Maillefer, Ballaigues, Switzerland) was placed in each canal until its tip was just visible at the root apex. Two periapical radiographs (mesiodistal and buccolingual) were taken for each root canal. Root canal curvatures were measured using Schneider’s method (15) and NIH Image-J software (version 1.52s; U.S. National Institutes of Health, Bethesda, Maryland, USA). After the measurement of the radius of curvature of each root canal, the canals were classified as slight (up to 5 degrees), moderate (from 5-20 degrees) and severe (greater than 25 degrees) curvature. All the root canals were distributed equally into three groups. There were no significant differences among the groups in terms of the curvatures (P>0.05). Each instrument was used in 3 root canals, 1 with a slight curvature and 2 with severe curvatures. The teeth were embedded in a sponge, and the preparations were performed at the simulated body temperature (37±0.5°C) (Fig. 1). The temperature was controlled using a thermostat. According to each instrument’s manufacturer recommendation, root canal instruments were operated using e Connect Pro (Changzhou Sifary Medical Technology Co. Ltd., Changzhou, China) endomotor. A single operator performed all the preparations. Only 1 rotary instrument was used for shaping the 3 root canals. The time (in seconds) needed for shaping the 3 root canals by each endodontic instrument during ex-vivo usage was recorded with an electronic stopwatch. The total time included instrumentation, instrument changes within the sequence, cleaning of the flutes of the instruments, and irrigation.

In group Ia, the coronal 2/3rd of the canals were scouted with a #10 K-file. Coronal flaring was performed by using TRN Orifice Modifier (#20/fixed 0.08 taper). TRN Glider (#17/average variable 0.02 taper) was advanced passively to the WL using gentle amplitudes. Canals were finally shaped with the TRN PRIME (#26/average variable 0.04 taper). All TRN instruments were used at the rotational speed of 500 rpm with a torque value of 150 Ncm.

In group Ib, the coronal 2/3^rd^ of the canals were scouted with a #10 K-file, and an HFC Orifice Opener (#25/.08) was used for coronal flaring. Next, HFC (#20/.04) was advanced into WL using the gentle amplitudes. The canals were finally shaped with the HFC (#25/.04). All HFC instruments were used at the rotational speed of 500 rpm with a torque of 250 Ncm.

In group Ic, the coronal 2/3^rd^ of the canals was scouted with a #10 K-file, and Endoflare Orifice Opener (#25/.12) was used for coronal flaring. G-file (#12/.03 & #17/.03) was used to establish the glide path. Canals were then shaped with the SC1(#25/.06) to 2/3^rd^ of working length in a free progression and without pressure. Subsequently, canals were prepared with an SC2 instrument (#25/.04) at the rotational speed of 300 rpm with a 150 Ncm while using a gentle up and down movement.

Irrigation was performed with 5.25% NaOCl (Chlor-Xtra, Vista Dental Products) using a 27-gauge needle (Dispovan, Hindustan Syringes and Medical Devices. Ltd., India) throughout the shaping procedure. The canals were recapitulated with #10 K-file between each instrument change. After every 3 in-and-out movements, the debris on the flutes of the instruments were cleaned. After the preparation, all used rotary files were inspected using an optical stereomicroscope (Lawrence and Mayo, London, England). However, none of the instruments broke. The coronal flaring and the glide path instruments were excluded from the cyclic fatigue testing as these instruments vary in their size and taper.

### d) Cyclic fatigue testing:

**Figure 2.**
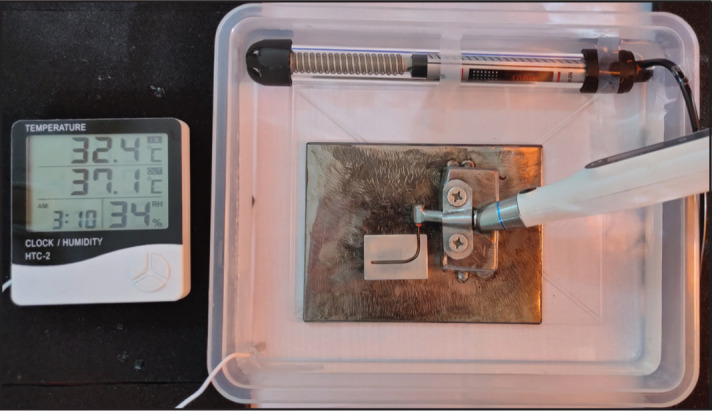
Cyclic fatigue testing device and attached endomotor assembly in a saline pool with a constant temperature (37±0.5°C)

A 316L stainless-steel metal block with an artificial canal with a 1.5 mm inner diameter was used for the test. Block’s coronal part was 7 mm (above the first point of the curvature part). The radius of curvature was 7 mm, and the angle was 90^°^. Glycerin (Delta Chemicals, Mumbai, India) was used as a lubricant. The block was immersed in a saline solution to simulate the body temperature (37±0.5°C) (Fig. 2). The temperature was controlled through a thermostat. Group I and group II instruments were tested at a constant length of 18 mm. Instruments were rotated at the speed and torque values according to the manufacturer’s instructions until a fracture occurred. A digital chronometer was used to record the time in seconds until the fracture occurred. A digital calliper was used to measure the fractured part’s length (in mm). The numbers of cycles to failure (NCF) was calculated according to the following formula (9):

NCF=rotational speed (rpm) × time to fracture (sec)/60

### Statistical analysis

Kolmogorov–Smirnov test with Lilliefors significance correction was performed to determine whether the data were normally distributed. The statistical analysis of stationary fatigue data, preparation time and length of fractured instruments were performed using two-way ANOVA and Tukey post-hoc tests at 95% confidence interval (α=0.05).

## Results

The observed NCF values were signiﬁcantly affected by the instrument type and an un-used or used variable (P<0.001). However, there was no signiﬁcant difference between the instrument type and unused or used variables (P>0.05) (Table 1). The difference in the cyclic fatigue resistance of the tested rotary files was statistically significant (P<0.05).

**Table 1.** Two-way ANOVA for the instrument type variable, unused or used variable and the interaction according to NCF data (P<0.05)

Source of variation	Mean squares	F	Partial eta squared	P-value*	Observed power
Instrument type variable	12194565.45	54.879	0.670	<0.001	1.000
Unused or used variable	3228651.248	14.53	0.212	<0.001	0.963
Interaction	421054.60	1.895	0.066	0.160	0.377

*Statistically signiﬁcant difference at P<0.05

Table 2 summarises the results of the stationary fatigue test. The unused (Group II) TRN, HFC & RS instruments were significantly more resistant to stationary fatigue as com-pared to the used ones (Group I) (P<0.05). In addition, the unused and used HFC instruments were more resistant to cyclic fatigue as compared to unused and used TRN and RS instruments (P<0.05) (Table 2). None of the endodontic instruments broke during the ex-vivo usage.

**Table 2.** Mean standard deviation of NCF registered during cyclic fatigue testing

Group	Condition	SD
TruNatomy	Unused	1825.0±231.6
TruNatomy	Used	1060.0±446.1
HyFlex	Unused	2297.5±958.5
HyFlex	Used	1856.7±367.3
Revo-S	Unused	617.0±118.9
Revo-S	Used	431.0±113.3

*Statistically signiﬁcant difference at P<0.05

The mean length (in mm) of fractured fragments was significantly (P=0.002) highest in the RS group, followed by HFC and TRN groups (Table 3). Also, the overall mean length of fractured fragments was significantly (P=0.001) higher amongst used as compared to unused instruments. The interaction effect of instrument type and usage (used/unused) of instruments was statistically significant (P<0.001), which means that both factors taken together significantly affect the mean length of fractured fragments. The highest mean length of fractured instruments was observed in RS used instruments (group Ic), followed by HFC used (group Ib), HFC unused (group IIb), TRN new (group IIa), TRN used (group Ia), and RS unused (group IIc) instruments. Regarding the time taken to prepare the canals, TRN instruments required significantly lesser time to prepare the canals as compared to HFC and RS instruments (P<0.05) (Table 4).

**TABLE 3.** Comparative assessment of length (mm) of fractured fragments registered during cyclic fatigue testing

Instrument type	Length (mm) of fractured fragments (Mean±SD)	P-value
	Used instrument	Unused instrument
TruNatomy	3.90±0.61	4.35±0.41	Main effect (Instrument type) 0.002*
HyFlex	4.50±0.81	4.30±0.48	
Revo-S	5.75±0.75	3.90±0.39	
P-value^#^	Main effect (Instrument usage) 0.001*	Interaction effect	<0.001

Test applied: Two-way ANOVA, SD: Standard deviation

**TABLE 4.** Mean preparation time (sec) and SD with the different instruments

Instrument	Mean	SD
TruNatomy (TRN)	638.2^a^	14.1
HyFlex CM (HFC)	726.2^b^	12.6
Revo-S (RS)	838.6^c^	23.7

*Values with the different superscript letters were statistically significant at P<0.05

## Discussion

This study evaluated the stationary cyclic fatigue of endodontic rotary instruments to demonstrate their performance and establish safety parameters for further clinical use. In the study, the difference in the cyclic fatigue resistance of the tested endodontic files was statistically significant (P<0.05). Thus, the null hypothesis was rejected.

Various factors affect the NiTi instrument behaviour during the endodontic shaping, such as design features, heat treatment, manufacturing process, and metallurgical differences (16, 17). The use of thermomechanical technology can improve the transformation characteristic and the microstructure of the NiTi instruments. The TRN and HFC instruments are thermomechanically treated NiTi files, and it influences the fatigue resistance, thereby improving the canal shaping performance of the instruments (18).

As anticipated, the unused (Group II) instruments were resistant to stationary fatigue compared to the used (Group I) ones. Unused TRN (group IIa) and Revo-S (group IIc) instruments were significantly more resistant to stationary fatigue as compared to the used ones (group Ia and group Ic). The reduction in resistance to stationary fatigue of used instruments was 41.9% for the TRN, 30.1% for the RS, and 19.2% for HFC. The reduction in resistance could be related to the fact that there remains no prior fatigue on unused instruments.

The unused and used HFC instruments were more resistant to cyclic fatigue as compared to unused and used TRN and RS instruments. The HFC instruments have a higher austenite finishing temperature (AF). Also, owing to its transition temperature between austenite and martensite phases, the instrument can remain in the stable martensitic phase at body temperature (19, 20). Therefore, the transition temperature might positively affect the stationary fatigue resistance of the HFC as the experiment was carried out at body temperature. In addition, the square cross-section design of HFC could contribute to higher cyclic fatigue resistance compared to the off-centred parallelogram of TRN and symmetrical cross-section of RS. The unused HFC and used HFC showed higher NCF, as compared to the unused and used RS instruments. The differences in NCF could be attributed to instrument design, alloy composition and thermal treatment.

The used and unused TRN and HFC instruments were more resistant to stationary fatigue than the used and unused RS instruments. The TRN and HFC instruments are manufactured from heat-treated alloy, unlike RS instruments manufactured from conventional NiTi alloy (10, 12). In addition, the alloy used for the HFC instrument has a lower percentage of nickel than the traditional NiTi alloys (52.1% in weight) (10). The alloy properties and the thermomechanical treatment of the instruments used can explain why the results differ from each other.

The unused and used TRN instruments were significantly more resistant to cyclic fatigue compared to the unused and used RS. This finding can be attributed to different NiTi diameters in manufacturing, metallurgical differences, cross-section design, and heat treatment (21). These may also explain the result of the low NCF of RS.

Capar et al. (11) observed lower cyclic fatigue resistance of RS than HFC, ProTaper Next and One Shape, and observed the role of traditional NiTi alloy in the manufacturing process. However, Moreinoset et al. (22) observed no significant difference between ProTaper Next (PTN) and RS. The difference in the methodology could be the reason for such a difference.

Pedullà et al. (23) and Lopes et al. (24) also reported the lowest cyclic fatigue resistance with the RS, compared to Twisted File, Mtwo, Biorace, K3XF, K3, and Profile Vortex.

In our research, HFC instruments were significantly more resistant to fracture than the TRN instruments. These results are in accordance with the study conducted by Gündoğar (13) and Elnaghy (2). In contrast, Elnaghy (7) observed no significant difference in the NCF between TRN and HFC. This can be attributed to differences in the methodology or study design.

The present study’s findings are in accordance with the study conducted by Capar et al. (11), who observed the highest fatigue resistance with HFC and lowest with the RS files. Unfortunately, there is scarce literature available comparing the cyclic fatigue resistance (CFR) of TRN and RS.

The highest mean length of separated instruments was observed in RS used instruments followed by HFC used, TRN unused, HFC unused, TRN used and RS unused instruments. Surprisingly, the unused category RS showed the least mean length. This could be related to the asymmetric cross-section design of the RS instruments that allowed minimal stress concentration along the instrument. However, in used instruments category, the TRN performed better owing that probably linked to regressive taper design and smaller file blank diameter. Preparation time depends on various factors such as the applied technique, the numbers of instruments used, and the operator’s experience (25). The RS instrument consumed significantly more time for canal preparation than the HFC and TRN. This could be attributed to the increasing number of instruments used. TRN was found to require significantly less time to prepare the canals as compared to HFC and RS.

Besides the metallurgy and manufacturing process, the instrument’s cyclic fatigue resistance is also affected by instrument design factors like tip design, diameter, cross-section, and taper (26). Furthermore, the speed of rotation and whether used in rotation or reciprocation may also determine the instrument’s fatigue resistance (27). The taper, tip diameter and manner of use of endodontic instruments in all the tested groups were comparable. Furthermore, to simulate the clinical condition, all the NiTi files were tested at standardised human body temperature. Studies have reported the ignorance of in-vivo intracanal temperature and the influence of ambient temperature on the cyclic fatigue resistance of thermomechanically treated NiTi files (28).

All the groups were standardised in the best possible way and used as per the manufacturer’s recommendations to minimise the risk of unreliable comparisons or bias among the tested groups. However, the present study lacked uniformity in glide file design and orifice shapers used. Hence, possible variations in the outcomes in used file groups are expected. Also, owing to the different designs and size of the coronal flaring and glide path instruments, the authors excluded them from the cyclic fatigue testing.

The experimental model of the present laboratory study lacked simulation of natural tooth structure. Also, the static cyclic fatigue testing did not simulate the clinical shaping procedure. Hence, further in-vivo studies are needed to investigate similar parameters in an actual clinical scenario.

## Conclusion

Based on the outcome of this research, it can be concluded that amongst the tested groups, HFC instruments were most resistant to cyclic fatigue, followed by TRN and RS instruments. Also, the TRN instruments took lesser time for the canal shaping as compared to HFC and RS instruments.

### Disclosures

**Conflict of interest:** The authors deny any conflicts of interest.

**Ethics Committee Approval:** Prior institutional Ethical Committee (PDCH/20/EC-228) approval was obtained for this *in-vitro* study.

**Peer-review:** Externally peer-reviewed.

**Financial Disclosure:** Nil.

**Authorship contributions:** Concept – A.A.; Design – A.A., N.U.G.; Supervision – N.C., A.A.; Funding - A.A.; Materials - A.A., N.U.G.; Data collection &/or processing – A.K.K., A.A., N.U.G.; Analysis and/or interpretation – A.K.K., A.A., N.U.G.; Literature search – A.A.; Writing – A.K.K., A.A., N.U.G.; Critical Review – A.K.K., A.A., N.U.G., N.C.

## References

[1] Shen Y, Cheung GS, Bian Z, Peng B (2006). Comparison of defects in ProFile and ProTaper systems after clinical use.. J Endod.

[2] Elnaghy AM, Elsaka SE, Elshazli AH (2020). Dynamic cyclic and torsional fatigue resistance of TruNatomy compared with different nickel-titanium rotary instruments.. Aust Endod J..

[3] Martín B, Zelada G, Varela P, Bahillo JG, Magán F, Ahn S (2003). Factors influencing the fracture of nickel-titanium rotary instruments.. Int Endod J.

[4] Peters OA (2004). Current challenges and concepts in the preparation of root canal systems: a review.. J Endod.

[5] Parashos P, Gordon I, Messer HH (2004). Factors influencing defects of rotary nickel-titanium endodontic instruments after clinical use.. J Endod.

[6] Subha N, Sikri VK (2011). Comparative evaluation of surface changes in four Ni-Ti instruments with successive uses - An SEM study.. J Conserv Dent.

[7] Elnaghy AM, Elsaka SE, Mandorah AO (2020). In vitro comparison of cyclic fatigue resistance of TruNatomy in single and double curvature canals compared with different nickel-titanium rotary instruments.. BMC Oral Health.

[8] Topçuoğlu HS, Topçuoğlu G, Kafdağ Ö, Balkaya H (2020). Effect of two different temperatures on resistance to cyclic fatigue of one Curve, EdgeFile, HyFlex CM and ProTaper next files.. Aust Endod J.

[9] Topçuoğlu HS, Topçuoğlu G, Akti A, Düzgün S (2016). In vitro comparison of cyclic fatigue resistance of ProTaper Next, HyFlex CM, OneShape, and ProTaper Universal instruments in a canal with a double curvature.. J Endod.

[10] Bürklein S, Börjes L, Schäfer E (2014). Comparison of preparation of curved root canals with Hyflex CM and Revo-S rotary nickel-titanium instruments.. Int Endod J.

[11] Capar ID, Ertas H, Arslan H (2015). Comparison of cyclic fatigue resistance of novel nickel-titanium rotary instruments.. Aust Endod J.

[12] Peters OA, Arias A, Choi A (2020). Mechanical properties of a novel nickel-titanium root canal instrument: stationary and dynamic tests.. J Endod.

[13] Gündoğar M, Uslu G, Özyürek T, Plotino G (2020). Comparison of the cyclic fatigue resistance of VDW.ROTATE, TruNatomy, 2Shape, and HyFlex CM nickel-titanium rotary files at body temperature.. Restor Dent Endod.

[14] Mustafa R, Al Omari T, Al-Nasrawi S, Al Fodeh R, Dkmak A, Haider J (2021). Evaluating in vitro performance of novel nickel-titanium rotary system (TruNatomy) based on debris extrusion and preparation time from severely curved canals.. J Endod.

[15] Schneider SW (1971). A comparison of canal preparations in straight and curved root canals.. Oral Surg Oral Med Oral Pathol.

[16] Shen Y, Zhou HM, Zheng YF, Peng B, Haapasalo M (2013). Current challenges and concepts of the thermomechanical treatment of nickel-titanium instruments.. J Endod.

[17] Park SY, Cheung GS, Yum J, Hur B, Park JK, Kim HC (2010). Dynamic torsional resistance of nickel-titanium rotary instruments.. J Endod.

[18] Hieawy A, Haapasalo M, Zhou H, Wang ZJ, Shen Y (2015). Phase transformation behavior and resistance to bending and cyclic fatigue of ProTaper Gold and ProTaper Universal instruments.. J Endod.

[19] Hou X, Yahata Y, Hayashi Y, Ebihara A, Hanawa T, Suda H (2011). Phase transformation behaviour and bending property of twisted nickel-titanium endodontic instruments.. Int Endod J.

[20] Santos Lde A, Bahia MG, de Las Casas EB, Buono VT (2013). Comparison of the mechanical behavior between controlled memory and superelastic nickel-titanium files via finite element analysis.. J Endod.

[21] Capar ID, Ertas H, Arslan H (2014). Comparison of cyclic fatigue resistance of nickel-titanium coronal flaring instruments.. J Endod.

[22] Moreinos D, Dakar A, Stone NJ, Moshonov J (2016). Evaluation of time to fracture and vertical forces applied by a novel gentlefile system for root canal preparation in simulated root canals.. J Endod.

[23] Pedullà E, Plotino G, Grande NM, Pappalardo A, Rapisarda E (2012). Cyclic fatigue resistance of four nickel-titanium rotary instruments: a comparative study.. Ann Stomatol (Roma).

[24] Lopes HP, Gambarra-Soares T, Elias CN, Siqueira JF, Inojosa IF, Lopes WS (2013). Comparison of the mechanical properties of rotary instruments made of conventional nickel-titanium wire, M-wire, or nickel-titanium alloy in R-phase.. J Endod.

[25] Lim KC, Webber J (1985). The validity of simulated root canals for the investigation of the prepared root canal shape.. Int Endod J.

[26] Ruiz-Sánchez C, Faus-Matoses V, Alegre-Domingo T, Faus-Matoses I, Faus-Llácer VJ (2018). An in vitro cyclic fatigue resistance comparison of conventional and new generation nickel-titanium rotary files.. J Clin Exp Dent.

[27] Pérez-Higueras JJ, Arias A, de la Macorra JC (2013). Cyclic fatigue resistance of K3, K3XF, and twisted file nickel-titanium files under continuous rotation or reciprocating motion.. J Endod.

[28] Yılmaz K, Uslu G, Gündoğar M, Özyürek T, Grande NM, Plotino G (2018). Cyclic fatigue resistances of several nickel-titanium glide path rotary and reciprocating instruments at body temperature.. Int Endod J.

